# Single-Cell Analysis Reveals the Immune Characteristics of Myeloid Cells and Memory T Cells in Recovered COVID-19 Patients With Different Severities

**DOI:** 10.3389/fimmu.2021.781432

**Published:** 2022-01-03

**Authors:** Xu Li, Manik Garg, Tingting Jia, Qijun Liao, Lifang Yuan, Mao Li, Zhengyu Wu, Weihua Wu, Yalan Bi, Nancy George, Irene Papatheodorou, Alvis Brazma, Huanle Luo, Shisong Fang, Zhichao Miao, Yuelong Shu

**Affiliations:** ^1^ School of Public Health (Shenzhen), Shenzhen Campus of Sun Yat-sen University, Shenzhen, China; ^2^ European Molecular Biology Laboratory, European Bioinformatics Institute (EMBL-EBI), Wellcome Genome Campus, Hinxton, Cambridge, United Kingdom; ^3^ Major Infectious Disease Control Key Laboratory, Shenzhen Center for Disease Control and Prevention, Shenzhen, China; ^4^ Translational Research Institute of Brain and Brain-Like Intelligence and Department of Anesthesiology, Shanghai Fourth People’s Hospital Affiliated to Tongji University School of Medicine, Shanghai, China

**Keywords:** memory T cells, HLA class II, recovered COVID-19 patients, disease severity, myeloid cells

## Abstract

Despite many studies on the immune characteristics of Coronavirus disease 2019 (COVID-19) patients in the progression stage, a detailed understanding of pertinent immune cells in recovered patients is lacking. We performed single-cell RNA sequencing on samples from recovered COVID-19 patients and healthy controls. We created a comprehensive immune landscape with more than 260,000 peripheral blood mononuclear cells (PBMCs) from 41 samples by integrating our dataset with previously reported datasets, which included samples collected between 27 and 47 days after symptom onset. According to our large-scale single-cell analysis, recovered patients, who had severe symptoms (severe/critical recovered), still exhibited peripheral immune disorders 1–2 months after symptom onset. Specifically, in these severe/critical recovered patients, human leukocyte antigen (HLA) class II and antigen processing pathways were downregulated in both CD14 monocytes and dendritic cells compared to healthy controls, while the proportion of CD14 monocytes increased. These may lead to the downregulation of T-cell differentiation pathways in memory T cells. However, in the mild/moderate recovered patients, the proportion of plasmacytoid dendritic cells increased compared to healthy controls, accompanied by the upregulation of *HLA-DRA* and *HLA-DRB1* in both CD14 monocytes and dendritic cells. In addition, T-cell differentiation regulation and memory T cell–related genes *FOS*, *JUN*, *CD69*, *CXCR4*, and *CD83* were upregulated in the mild/moderate recovered patients. Further, the immunoglobulin heavy chain V3-21 (*IGHV3-21*) gene segment was preferred in B-cell immune repertoires in severe/critical recovered patients. Collectively, we provide a large-scale single-cell atlas of the peripheral immune response in recovered COVID-19 patients.

## Introduction

Coronavirus disease 2019 (COVID-19), caused by severe acute respiratory syndrome coronavirus 2 (SARS-CoV-2), has resulted in more than 261 million confirmed cases and more than 5.2 million deaths according to the statistics of the World Health Organization (WHO) as of November 30, 2021. The impact of this disease has led to extensive research work to quickly understand, control, and treat COVID-19. Most previous single-cell studies (1–13) focused on the COVID-19 progression stage and have provided important immune cellular and molecular characteristics. The large-scale integrated analysis of single-cell data by Ren et al. (9) included single-cell sequencing data of 140 different types of samples from 104 COVID-19 convalescent patients, showed the immune cell proportions of peripheral blood mononuclear cells (PBMCs) and T-cell receptor (TCR) clone diversity and other characteristics in convalescent patients. Zhang et al. (8) profiled adaptive immune cells of PBMCs from recovered COVID-19 patients with varying disease severity using single-cell RNA sequencing (scRNA-seq), single-cell TCR sequencing (scTCR-seq), and single-cell BCR sequencing (scBCR-seq). However, these studies cannot explain the phenomenon recently discovered by Amanat et al. (14) that convalescent patients with high serum anti-spike titers produce a higher proportion of non-neutralizing antibodies. Data from several studies suggested that patients with severe COVID-19 had higher serum anti-spike titers (15–18). In addition, according to a large-scale population survey in Denmark, the protection rate of individuals under 65 years of age against SARS-CoV-2 reinfection is higher than 80%, while patients 65 years of age and older have only 47.1% protection (19). There is currently no clear explanation for this. Hence, there is an urgent need for a deeper and more comprehensive understanding of the immune characteristics of COVID-19 patients during the recovery stage.

Here we analyzed the scRNA-seq data of 41 individuals of more than 260,000 cells, including 16 mild/moderate recovered patients, 6 severe/critical recovered patients, and 19 healthy controls. CD14 monocytes (CD14 mono), CD4 T cells, and CD8 T cells in severe/critical recovered patients were still in a disordered state 27–47 days after symptom onset, accompanied by a high expression of cytokines and interferon-stimulated genes (ISGs). The percentages of CD4 T cells and CD8 T cells in mild/moderate recovered patients were comparable to healthy controls, but showed significant transcriptome changes. Our data and findings may have important implications for revealing the relationship between the immune response of patients recovering from COVID-19 and the immune protection against SARS-CoV-2 reinfection.

## Materials and Methods

### PBMCs From Blood

The dataset generated in this study was termed as the “Li dataset”. To generate this dataset, human blood samples were collected by Shenzhen Center for Disease Control and Prevention, Shenzhen, China. We collected PBMC samples at 27–47 days after onset of symptoms or tested with SARS-CoV-2 nucleic acid positive. All patients were in recovery stage or had no clinical symptoms at sample collection (**Table S1A**). PBMCs were isolated immediately, using lymphocyte separation fluid under the enhanced biosafety level 2 facility. Then, we used a freshly prepared freezing solution [fetal bovine serum (FBS) containing 10% dimethyl sulfoxide (DMSO)] to freeze the PBMCs. In addition, we collected two fresh PBMC samples from two healthy individuals for sequencing as healthy control (**Table S1A**).

### Single-Cell 5′ mRNA and VDJ Sequencing

For the Li dataset, after sample collection, the PBMCs were stored in liquid nitrogen. Cell suspensions were barcoded through the 10x Chromium Single Cell platform using the Chromium Single Cell 5′ Library, Gel Bead and Multiplex, and Chip kits (10x Genomics). Twenty thousand PBMCs were loaded on each 10X Chromium A Chip. Single-cell lysis and RNA first-strand synthesis were performed using the 10X Chromium Single Cell 5′ Library and Gel Bead Kit according to the manufacturer’s protocol. RNA and VDJ library preparation were performed according to the manufacturer’s instructions, using the Chromium Single Cell 5′ v3 Reagent (10x Genomics) and Chromium Single Cell V(D)J Reagent kits (10x Genomics). Each sequencing library was generated with a unique sample index. All libraries were quantified by Qubit 3.0 (Thermo Fisher), Agilent 2100, and Qsep 100. Sequencing was performed on a Hiseq4000 platform with a paired-end 150 sequencing strategy.

### Single-Cell RNA-Seq Data Preprocessing

For the Li dataset, we used the Cell Ranger single-cell software suite (version 3.0.0, 10x Genomics) to compare and quantify the single-cell sequencing data against the GRCh38 human reference genome. Firstly, the cells in each sample were screened, and cells expressing at least 200 genes were kept. Next, cells were filtered according to three criteria: (1) cells must have a proportion of mitochondrial gene counts (UMIs from mitochondrial genes/total UMIs) of less than 15%; (2) cells must have a total number of unique molecular identifiers (UMI) counts per cell (library size) of more than 500; (3) genes must be expressed in more than two cells. Doublets were identified using Scrublet (20) and were removed from the analysis. After quality control filtering, a total number of 86,650 cells were retained for downstream analysis (details are shown in **Table S2A, B**).

For the preprocessing of the Ren dataset (9), we downloaded the scRNA-seq expression profile from NCBI Gene Expression Omnibus (GEO) database: GSE158055 (https://www.ncbi.nlm.nih.gov/geo/query/acc.cgi?acc=GSE158055). Only healthy control samples and samples of 27 and 47 days after recovery (hospital discharge) were included in our analysis. As the downloaded data had already been analyzed and annotated, all the cells in the dataset were used in our analysis without quality control. The two datasets were merged together based on the raw counts using the concatenate function in Scanpy (21). An overlap of 22,094 genes was found in the merged data. To integrate the Li dataset and the Ren dataset, we selected the top 2,000 highly variable genes for the integrated dataset using the “seurat_v3” flavor in the scanpy.pp.highly_variable_genes() function in Scanpy (21).

### Single-Cell RNA-Seq Cell Type Annotation

Scanpy (21) was used to analyze the data, including normalization, transformation, highly variable gene selection, and dimension reduction. The expression profile was first normalized to counts per ten thousand (CPTT) by scanpy.pp.normalize_total() function and then log-transformed by scanpy.pp.log1p(). Highly variable genes were selected with scanpy.pp.highly_variable_genes() according to the mean expression and dispersion of the genes. Principal Component Analysis (PCA) was performed on the expression profile of the highly variable genes. Harmony (version 0.0.5) (22) was used to integrate data on the latent space from different samples on the PCA space. Dimension reduction was performed with Uniform Manifold Approximation and Projection (UMAP) (23). To present cell clustering using Louvain method (24) on the Harmony corrected latent space. The cluster stabilities were assessed by self-projection (25). A machine learning-based method (25) was used to infer cell types according to two annotated reference datasets (3, 6).

### Immune Cell Proportion Analysis

We calculated immune cell proportions for each major cell type and cell subtype. For each sample, cell type proportion was calculated by the number of cells in a certain cell type divided by total number of cells. To identify changes in cell proportions between samples in different disease severity states and sex, we performed T tests and non-parametric tests on the proportions of each major cell type and cell subtype across different groups. We performed Spearman’s correlation analysis to assess the association between cell type proportion and patient age. A value of p less than 0.05 is regarded as significant.

### ANOVA and Linear Regression Analysis

To further evaluate the influence of different sample technical factors, patient phenotypes, and their potential interactions to cell type proportions, we performed One-way ANOVA on cell type proportions based on different phenotypes (**Figures S2A**), including disease severity, sex, and sample type (fresh or frozen). In **Figure S2A**, we included the sample data from the Ren dataset with 21–90 days sampling time (days after symptom onset) for One-way ANOVA and linear regression analysis. Cell type proportions were used as the outcome in a regression analysis with age and sampling time (days after symptom onset) as predictors, respectively. Following a multiple testing correction, phenotypes were regarded as significantly associated with cell type proportions when q value is less than 0.05. Statistical analyses were performed using R software (v 4.1.1; The R Foundation).

### Differential Expression and Kyoto Encyclopedia of Genes and Genomes Analysis

To model the differential expression of genes between cells together with technical effects, we used the hurdle model in MAST (26), while accounting for the covariates of sex and age. In the model, the effects of covariates are regressed out such that the differential expression represents the effect of the disease condition. Differentially expressed genes with a false detection rate (FDR) lower than 0.01 were used for volcano plots and pathway/gene ontology analysis. Upregulated genes were defined as the ones with a positive log fold change value. GProfiler (27) was used to analyze and visualize the regulated pathways based on the Gene Set Enrichment Analysis (GSEA) database of hallmark genes (28), while the Kyoto Encyclopedia of Genes and Genomes (KEGG) hallmark gene set was used in the analysis.

### Gene Module Score Calculation

The gene module score was calculated as the average expression of a set of genes in a given gene module subtracted with the average expression of a reference set of genes. The latter were randomly sampled from the gene_pool for each binned expression value. For a given set of genes belonging to each module (such as HLA class II, cytokine module), the scores were generated using scanpy.tl.score_genes() function of Scanpy (v1.8.1) (21) with the parameter ctrl_size=100.

### Single-Cell TCR/BCR Analysis

The Ren TCR/BCR data (9) were first downloaded from NCBI Gene Expression Omnibus (GEO) database: GSE158055 (https://www.ncbi.nlm.nih.gov/geo/query/acc.cgi?acc=GSE158055). These data have been preprocessed by Ren et al. (9), and only those T cells with at least one TCR α-chain (TRA) and one TCR β-chain (TRB) were provided in this dataset. Similar preprocessing was done for BCR data where cells with at least one heavy chain (IGH) and one light chain (IGK/IGL) were provided. The clonotype frequencies for each sample were also provided. We preprocessed our TCR/BCR datasets in a similar manner before integrating them with Ren’s dataset and calculated the clonotype frequencies per sample. Please note that only those B-cell/T-cell clones with corresponding cell-type annotations obtained from scRNA-seq analysis (see **Methods** section *Single-Cell RNA-Seq Cell Type Annotation*) were considered for further analysis. The final numbers are given in **Table S2A**. The UMAPs were plotted using the scanpy.pl.umap() function in (21), and the rest of the analysis was done using R-package Immunarch (v0.6.6) (29, 30).

### Cell–Cell Interaction Analysis

The Scanpy AnnData (21) containing all cells was subsetted to T cells, Natural killer (NK) cells, monocytes, dendritic cells (DCs), and plasmacytoid dendritic cells (pDCs) belonging to the 41 samples with a sampling date 27–47 days. This AnnData was further divided into three subsets corresponding to the three severities. The R-package CellChat (v1.1.3) (31) was used to infer cell–cell interaction networks where these annData objects used by Scanpy were converted to separate cellchat objects and then merged for comparison. To identify the upregulated and downregulated signaling pathways using differential expression analysis, two cellchat objects were analyzed at a time. Those differentially expressed signaling ligands with a Bonferroni corrected p-value lower than 0.05 and a log fold change higher than 0.01 were considered upregulated in the second dataset, while those ligands and receptors with a Bonferroni corrected p-value lower than 0.05 and a log fold change higher than 0.01 in the first dataset were considered downregulated in the second dataset. Only those ligand/receptor genes expressed in at least 10% of cells in the respective datasets were considered for visualization and analysis.

## Results

### The Recovered COVID-19 Patient Cohorts

**Figure 1:**
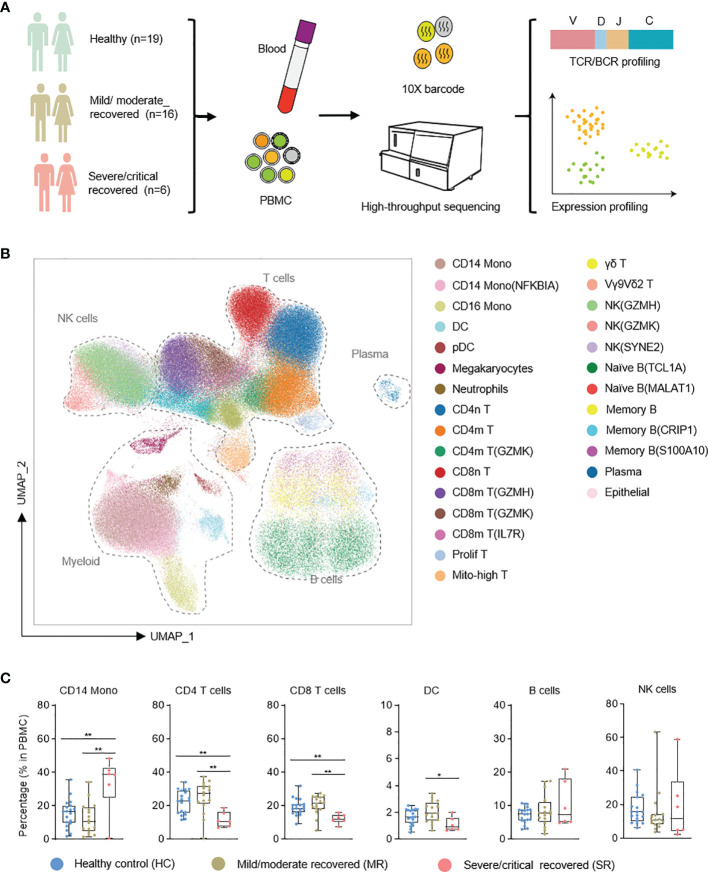
Single-cell atlas of recovered COVID-19 patients and healthy controls. **(A)** Flowchart depicting the overall design of the study. **(B)** UMAP presentation of the integrated single-cell transcriptomes of cells derived from recovered COVID-19 patients and healthy controls. **(C)** Box plots show the comparative analysis of the percentage of major cell types in PBMC cells. NK, natural killer cells; Mono, monocytes; DC, dendritic cells. T test with healthy, *p < 0.05, **p < 0.01.

To profile the transcriptional immune landscape of COVID-19 patients in the recovery stage, we collected 10 PBMC samples from recovered COVID-19 patients 27–47 days after symptom onset, and 2 PBMC samples from healthy control. Using single-cell sequencing technologies, we performed single-cell RNA sequencing as well as single-cell immune profiling (both single-cell B-cell receptor sequencing, scBCR-seq, and single-cell T-cell receptor sequencing, scTCR-seq) on these 12 samples (we term the dataset we generated as the “Li dataset”; see **Methods**). To improve the reliability and reproducibility of the data analysis, we added 12 PBMC samples from COVID-19 patients in the recovery stage from the previously reported dataset (9) along with 17 PBMC samples from healthy control (this added dataset is termed as the “Ren dataset”). These samples were selected to match the sampling time (according to the day after symptom onset) of the Li dataset, *i.e.*, both datasets had a sampling day of 27–47 days after symptom onset (**Tables S1A, B**). The overall integrated data included 19 healthy control samples (HC), 16 mild/moderate recovered (MR) samples, and 6 severe/critical recovered (SR) samples, which were classified according to WHO criteria (https://www.who.int/publications/i/item/covid-19-therapeutic-trial-synopsis) (**Figure 1A** and **Tables S1A, B**). In **Table S1C**, we compare the detailed characteristics of patients and controls (including median sampling day and sample type, patient median age, gender, comorbidities, and outcome). Consistent with other reports (9, 16), we found that the median age of severe/critical patients is greater than that of mild/moderate patients. The median sampling day of the mild/moderate and severe/critical recovery groups are 33.5 and 34.5 days (days after symptom onset), respectively (**Table S1C**). This basically eliminates the effect of detection time on the result of scRNA-seq of patients, and these two groups are comparable in our data.

After quality control, we obtained transcriptomes of 86,650 cells, 3,693 productive BCR clones, and 15,717 productive TCR clones from the Li dataset (**Table S2A**). From the Ren dataset we retrieved 178,239 cells, 13,899 productive BCR clones, and 41,676 productive TCR clones (**Table S2A**). A Unified Manifold Approximation and Projection (UMAP) based on the Harmony-corrected latent space was generated (**Figure 1B**; see **Methods**). We identified 28 distinct cell populations using a machine learning-based approach (25) in comparison with two annotated reference datasets (3, 6) (**Figure 1B**). These cell populations were further confirmed with known marker gene expression (**Figures S1A, B**).

We first analyzed the compositional changes of the broad categories of immune cells in PBMC (**Figure 1C**). Notably, in severe/critical COVID-19 patients in the recovery stage, the proportion of CD14 monocytes (based on the expression of the marker genes CST3, LYZ, CD14) in PBMCs were elevated, but CD4 T cells (IL7R, LTB) were decreased (**Figure 1C**), consistent with a previous report (9). CD8 T cells (which express CD8A, CD8B) decrease in severe/critical recovered patients in comparison with healthy controls.

### Immune Characteristics of Myeloid Cells in the Recovery Stage

**Figure 2:**
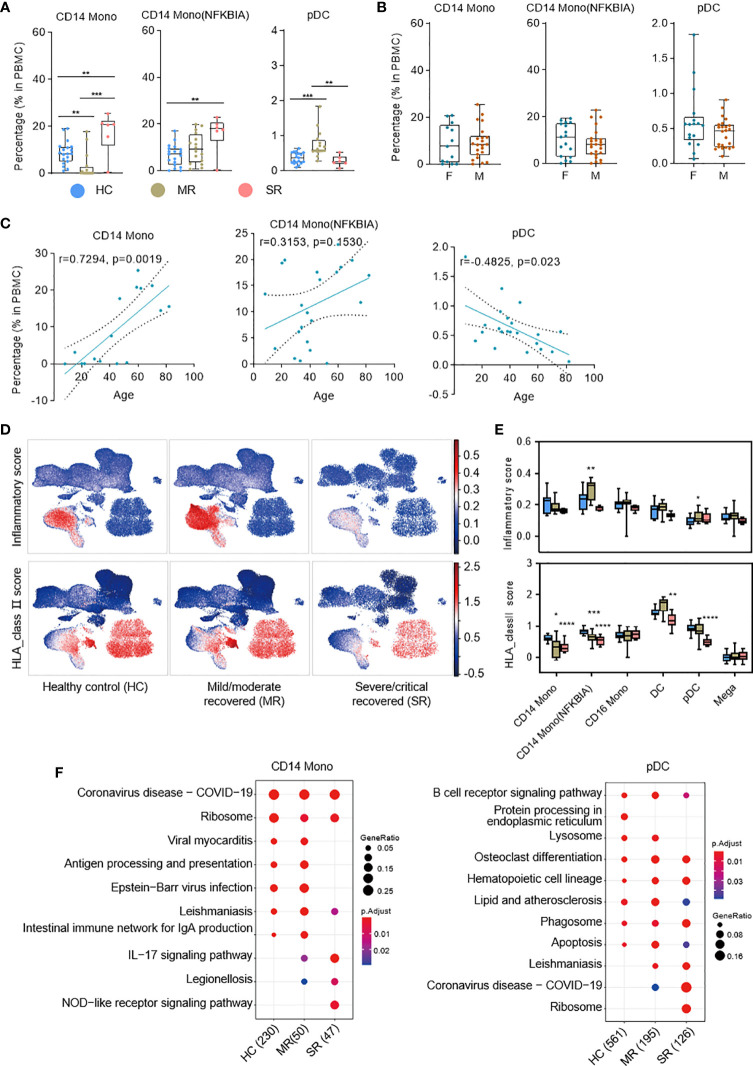
Single-cell transcriptome characteristics of the myeloid immune response in recovered COVID-19 patients. **(A)** Boxplots depicting percentages of multiple cell types in PBMC cells, colored by group-specific color. T tests (and non-parametric tests), *p < 0.05, **p < 0.01, ***p < 0.001, ****p < 0.0001. **(B)** The box plots show the cell subtype proportions in different genders. **(C)** The correlation analysis charts show the correlation between patient age and cell subtype proportions (Spearman’s correlation). **(D)** UMAPs of PBMC cells colored by inflammatory score (top panel) and HLA_class II score (bottom panel). **(E)** Box plots show the inflammatory score (top panel) and HLA_class II score ​(bottom panel) of subtypes from healthy controls (n = 19), mild/moderate recovered (n=16), severe/critical recovered (n=6) patients. Significance was evaluated with T tests (and non-parametric tests), for each subtype *versus* healthy controls. **(F)** Dot plots depict enriched signaling pathways in different serious groups in CD14 monocytes and pDCs. The number in parentheses represents the number of genes with significant differences. Mono, monocytes; pDCs, plasmacytoid dendritic cells; HC, healthy control; MR, mild/moderate recovered; SR, severe/critical recovered. T tests (and non-parametric tests), *p < 0.05, **p < 0.01, ***p < 0.001, ****p < 0.0001.

Multiple subtypes of myeloid cells significantly changed in cell proportions and genes transcription during the progression of COVID-19 dependent on symptom severity (6, 9). To understand the immune characteristics of these myeloid cell subtypes during the recovery stage of COVID-19, we analyzed the relationship between patient age, sex, symptom severity, and PBMC compositions (**Figure 2** and **Figures S2**, **S3**). The percentages of CD14 Mono and CD14 Mono (NFKBIA) cells were significantly higher in severe/critical recovered patients compared with healthy controls. We analyzed the correlation between all COVID-19 convalescent patient samples and age, and found CD14 Mono increases with age in the convalescent COVID-19 patients (r=0.7294, p=0.0019), but CD14 Mono (NFKBIA) has no significant correlation with age (r=0.3153, p=0.1530) (**Figure 2C**). Sex has no significant effect on the proportions of these two cell subtypes (p>0.05) (**Figure 2B**). Surprisingly, pDCs were significantly elevated in mild/moderate recovered patients but were comparable with healthy controls in severe/critical recovered patients (**Figure 2A**). Sex has no significant effect on the percentage of pDCs, but age is negatively correlated with the percentage of pDCs (r=−0.4825, p=0.023) (**Figures 2B, C**).

Next, we defined an inflammatory score and HLA class II score for each cell based on the expression of the reported inflammatory response genes (32) and HLA class II genes (6) (**Table S3**), respectively. We used these two scores to evaluate the inflammation status and antigen presentation ability for each cell (**Figures 2D, E**). The expression of HLA class II genes was significantly reduced in severe/critical recovered patients, and this reduction was more significant in monocyte and dendritic cell populations (**Figures 2D, E**). Similarly, we calculated the cytokine score and ISG score of each cell according to the expression of the collected cytokine genes and ISG genes (**Table S3**; see **Methods**). The expression of genes in cytokine module was significantly increased in severe/critical recovered patients but was comparable to healthy controls in mild/moderate recovered patients or showed a downward trend (**Figures S2B, C**). This phenomenon was observed in most cell subtypes. The expression of genes in the ISG module was essentially restored to healthy levels in recovered COVID-19 patients, but remained high in a subset of cell subtypes in severe/critical recovered patients, for example Prolif T, CD8m T(GZMH), and NK(GZMH) (**Figures S2B, C**).

Through the KEGG (33) pathway analysis, we further detected the differences in cell function of CD14 Mono, CD14 Mono (NFKBIA), and pDCs in recovered patients with different clinical severity (**Figure 2F** and **Figure S2D**). In CD14 monocytes, the nucleotide-binding oligomerization domain (NOD)-like receptor signaling pathway was enriched in severe/critical recovered patients, but the antigen processing and presentation and Intestinal immune network for IgA production were downregulated (**Figure 2F**). In pDCs, Lysosome pathway was downregulated in severe/critical recovered patients. Together with the significant decrease in HLA class II score, it suggested that the severe/critical recovered patients’ antigen processing and presentation ability was reduced (**Figures 2E, F**).

### Single-Cell Transcriptional Landscape of T Cells

**Figure 3:**
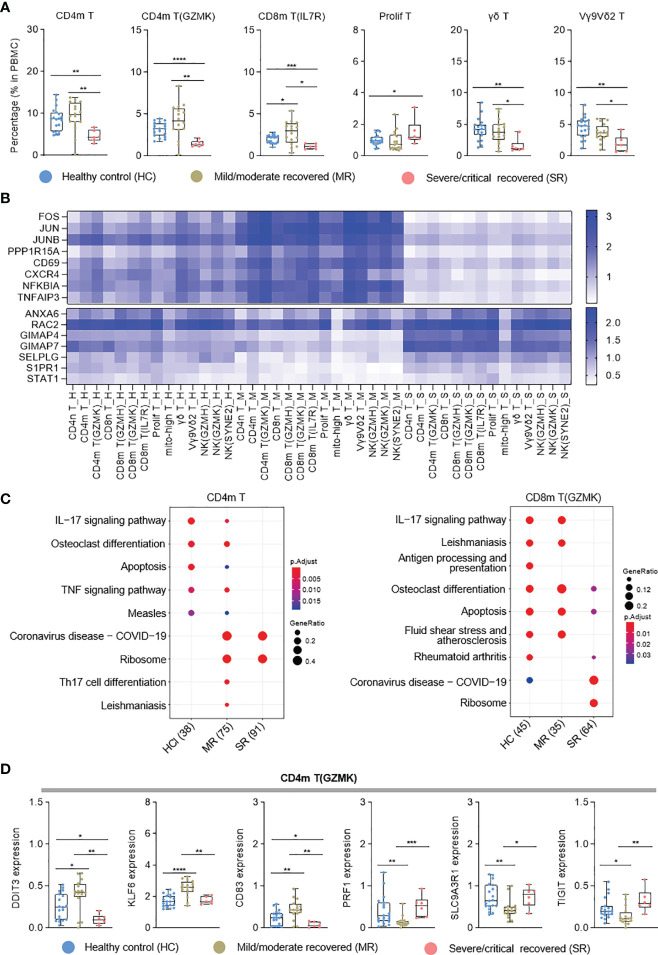
Single-cell transcriptome characteristics of the T and NK cell immune response in recovered COVID-19 patients. **(A)** Boxplots depicting percentages of T cell subtypes in PBMC cells, colored by group-specific color. **(B)** Heatmap visualization of average mRNA expression levels of the differential genes in three severity groups in T and NK cell subtypes. **(C)** Dot plot depicting enriched signaling pathways in different serious groups in CD4m T and CD8m T(GZMK). HC, healthy control; MR, mild/moderate recovered; SR, severe/critical recovered. The number in parentheses represents the number of genes with significant differences. **(D)** Boxplots of the gene expression of CD4m T(GZMK) cluster from healthy controls (n = 19), mild/moderate recovered (n = 16), severe/critical recovered (n = 6) patients. T tests (and non-parametric tests), *p < 0.05, **p < 0.01, ***p < 0.001, ****p < 0.0001.

T-cell immunity plays an important role in COVID-19 patients (4). To further clarify the T-cell immune characteristics of COVID-19 patients during the recovery stage, we analyzed each T-cell subtype. According to the cell proportion analysis, CD4m T, CD4m T (GZMK), CD8m T (IL7R), γδ T, and Vγ9Vδ2 T (34, 35) cells significantly decreased in severe/critical recovered patients, but were comparable with healthy controls in mild/moderate recovered patients (**Figure 3A** and **Figure S3A**). Unlike these T-cell subtypes, proliferating T (prolif T) cells were increased in severe/critical recovered patients compared to healthy controls (**Figure 3A**), which was consistent with the results reported by Ren et al. (9). In the correlation analysis with age, prolif T cells were positively correlated with age (r=0.5206, p=0.0005), while γδ T cells and Vγ9Vδ2 T cells were negatively correlated with age (r=−0.4498, p=0.0032; r=0.4344, p =0.0045, respectively) as shown in **Figures S3B, C**. Similar to myeloid cells, sex had no significant effect on all T-cell subtypes (data not shown).

Interestingly, in NK and T cell types, the transcriptional activator genes and functional immune genes, such as *FOS*, *JUN*, *CD69*, *CXCR4*, *NFKBIA* and *TNFAIP3*, were generally elevated in mild/moderate recovered patients (**Figure 3B**), while the expression of *GIMAP4*, *SELPLG*, *S1PR1*, and *STAT1* genes decreased (**Figure 3B**). In the KEGG pathway analysis, CD4m T cells exhibited enriched Th17 cell differentiation in mild/moderate recovered patients, while severe/critical recovered patients lacked IL-17 and TNF signaling pathway (**Figure 3C**). IL−17 signaling pathway was also downregulated in severe/critical recovered patients for CD8m T (GZMK) (**Figure 3C**). In CD4m T (GZMK), the cytotoxic activity-related *PRF1* and *SLC9A3R1* gene expression level was significantly reduced, *KLF6* and *CD83* were significantly increased in mild/moderate recovered patients, and the expression level of these genes in severe/critical recovered patients was comparable to healthy controls (**Figure 3D**). These four functional genes had similar expression pattern in the different severity for three CD8 memory T cell types (**Figure S3D**). *IFNG* was significantly increased in the mild/moderate recovered patients for the three CD8 memory T cell types (**Figure S3D**). These results suggested that the percentages of CD4 T cells and CD8 T cells was significantly reduced in severe/critical recovered patients, but the expression level of transcriptional activator genes and functional immune genes was comparable to healthy controls or had a slightly downward trend. Although the percentage of CD4m T, CD4m T (GZMK), and three CD8 memory T cell types in mild/moderate recovered was equal to or slightly higher than that in healthy controls. The memory T cells differentiation-related genes were significantly upregulated. These different immune characteristics may produce different SARS-CoV-2-specific memory T cells in different recovered patients.

### T-Cell and B-Cell Immune Repertoires in Recovered COVID-19 Patients

**Figure 4:**
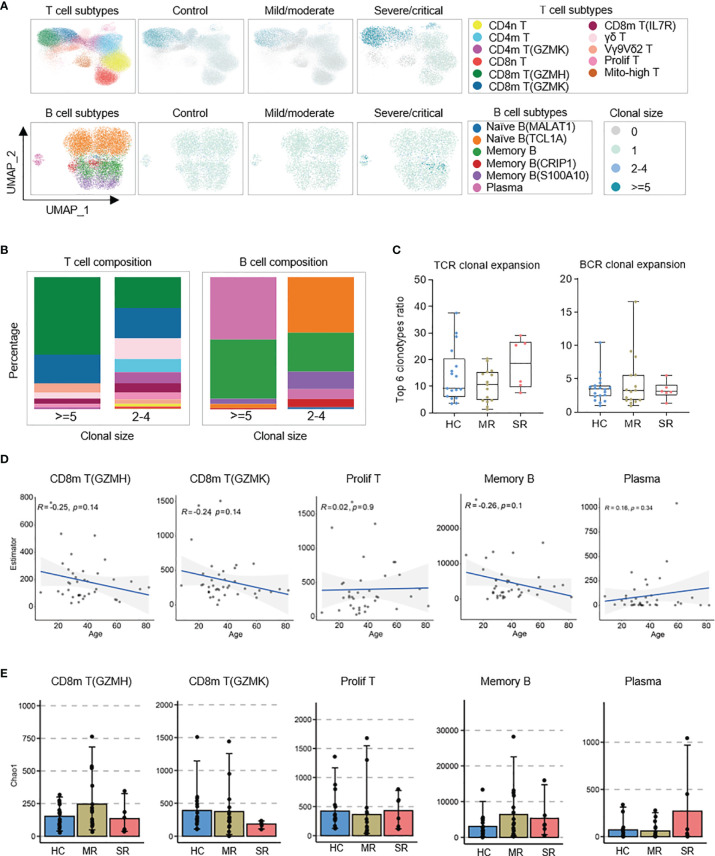
Distribution of BCR/TCR expanded clonotypes and associations of patient age and COVID-19 severity with the diversity of B and T cell repertoires. **(A)** UMAPs embedding of T/B cells colored by the density of cells characterized by different clonal expansion sizes (n = 1, n = 2-4, and n > = 5). Shown separately in different COVID-19 severity. **(B)** Column charts of T/B cell subpopulation composition of expanded TCR/BCR clones. **(C)** Box plots show characterization and comparison of TCR clonal expansion among severe/critical recovered patients (SR), mild/moderate recovered patients (MR), and healthy controls (HC), by quantifying the ratio of expanded clones. **(D)** The correlation analysis charts show the correlation between patient age and the BCR/TCR diversity of CD8m T(GZMH), CD8m T(GZMK), Prolif T, Memory B, and Plasma (Spearman’s correlation). **(E)** Box plots show the BCR/TCR diversity of CD8m T(GZMH), CD8m T(GZMK), Prolif T, Memory B, and Plasma among severe/critical recovered patients (SR), mild/moderate recovered patients (MR), and healthy controls (HC). The chao1 method in R-package Immunarch was used to evaluate repertoire diversity.

Our sequencing data also included scTCR-seq and scBCR-seq dataset to investigate the characteristics of TCR/BCR immune repertoires in recovered COVID-19 patients. UMAP results showed the distribution of TCR/BCR clone size in T/B cell subpopulations. The expanded clonotypes of the TCR clonal size >=5 were mainly from CD8m T (GZMH), CD8m T (GZMK), and Vγ9Vδ2 T cells (**Figures 4A, B**). Also, the expanded BCR clonotype ratio was very small. The clonotypes of BCR clonal size >=5 mainly came from plasma and memory B cells, and the clonotypes of BCR clonal size 2–4 mainly came from naïve B(TCL1A) and memory B cells (**Figures 4A, B**). Surprisingly, we found comparable T-/B-cell clonal expansion in healthy controls, mild/moderate, and severe/critical recovered patients (**Figure 4C** and **Figure S4A**). This was not consistent with the results reported by Zhang et al. (8). Whether this inconsistency was caused by the difference of sample types (the sample types used by Zhang et al. were peripheral blood CD3+ T cells and CD3−CD19+CD20+CD27+ antigen-experienced B cells) or the number of productive TCR/BCR clones obtained by sequencing or number of samples, it needs to be further verified. Further, in these cell types, clinical severity did not affect the TCR/BCR diversity (**Figure 4E**). The TCR/BCR diversity of CD8m T (GZMH), CD8m T (GZMK), and memory B was negatively correlated with age (R<0), while that of prolif T and plasma had a positive trend with age with no significant (p>0.05; **Figure 4D**). In addition, sex does not affect the TCR/BCR diversity of these cells (**Figure S4B**).

Next, to reveal the unique gene patterns and preferences of BCR or TCR in recovered COVID-19 patients, we compared the usage rate of immunoglobulin variable (V) genes. The severe/critical recovered patients use IGHV3-21 more frequently, compared to healthy controls (**Figure S4C**). Patients in the recovery stage had a certain bias towards the usage of other V genes as well, but there were large variations among individuals, and there was no statistically difference (**Figure S4C**). Finally, we analyzed the distribution of the heavy chain CDR3 (HCDR3) amino acid sequence length in the BCR repertoires of memory B cells. There was no significant difference in the distribution of HCDR3 lengths in memory B cells between the recovery stage of COVID-19 and the healthy control (**Figure S4D**).

### The Cross-Talk Between Myeloid Cells and T-Cells in the Recovered COVID-19 Patients

**Figure 5:**
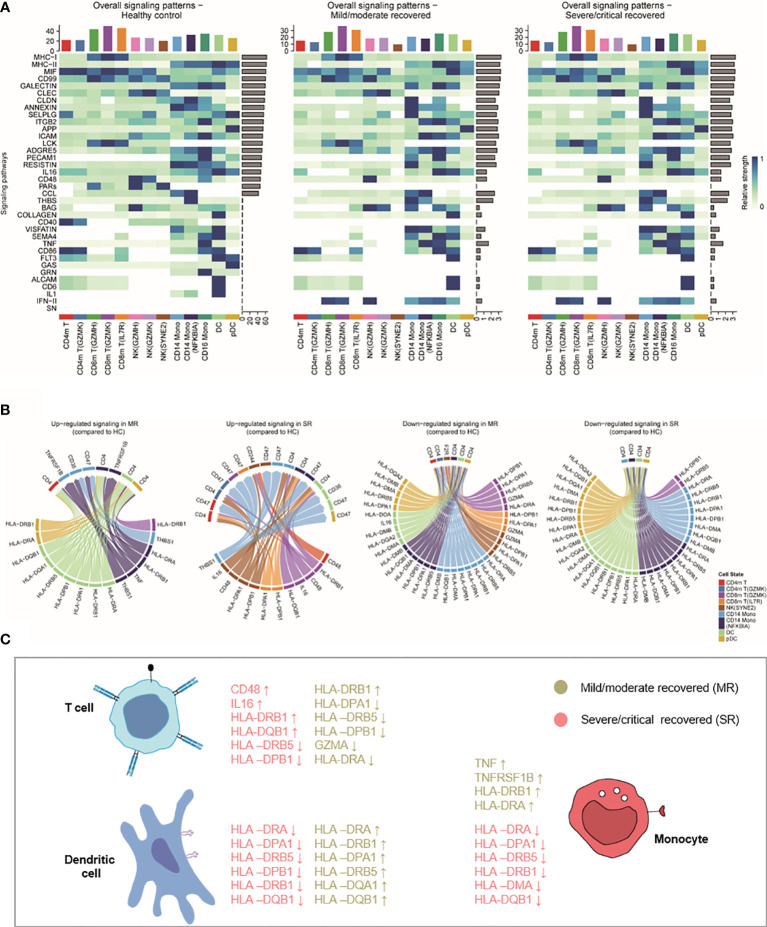
The interactions of monocyte, DC, NK, and CD4+/CD8+ memory T cell. **(A)** Heatmaps showing the overall signaling associated with each cell subtype. ​For each signaling pathway considered for the cell–cell interaction analysis using CellChat (see **Methods**), we can compare the aggregated incoming and outgoing signaling for each cell subtype in each severity. The top barplot represents the total non-normalized signaling for each cell subpopulation, while the right barplot represents the total log-normalized signaling for each pathway. **(B)** Circos plot showing the prioritized interactions mediated by ligand-receptor pairs between different cell types. HC, healthy control; MR, mild/moderate recovered; SR, severe/critical recovered; DC, dendritic cells; Mono, monocytes; NK, natural killer cells. **(C)** Summary illustration comparing the list of HLA genes and inflammatory genes that were upregulated or downregulated in mild/moderate recovery (MR) and severe/critical recovery (SR) compared to healthy controls (HC) in T cells, DCs, and monocytes.

In myeloid cell subtypes, the HLA class II score significantly decreased in CD14 monocytes and DCs of severe/critical recovered patients. We also found that the antigen processing or presentation pathways of CD14 monocytes and pDCs were downregulated in severe/critical recovered patients. We further investigated whether these changes contributed to the differences in CD4m T and CD8m T cell proportions, and cell–cell interaction analysis on the main subgroups of monocytes, DCs, and CD4m T cells and CD8m T cells was performed (**Figure 5**; see **Methods**).

For each signaling pathway considered for the cell–cell interaction analysis (see **Methods**), we compared the aggregated incoming and outgoing signaling for each cell population. While mild/moderate recovered and severe/critical recovered patients presented similar overall signaling patterns with different patterns to healthy controls (**Figure 5A**). Based on these signaling patterns, we focused on the interaction of MHC-II, TNF, IL1, IL16, INF-II, and CD48 and other ligand-receptor pairs in monocytes, DCs, and CD4 and CD8 memory T cells (**Figures 5B, C**). In the Circos plot, we found in an unbiased manner that the MHC II signaling was downregulated in CD14 monocytes, DCs, pDCs in severe/critical recovered patients compared to healthy controls. The TNF_TNFRSF1B ligand-receptor pair related to inflammation expression was upregulated in mild/moderate recovered patients compared to healthy controls (**Figure 5B**). These results were consistent with our previous observations with HLA class II score and inflammatory score trends (**Figures 2D, E**). Surprisingly, *HLA-DRA*, *HLA-DRB1*, and other HLA class II genes were downregulated in CD14 monocytes, DCs, pDCs in severe/critical recovered patients, but they were upregulated in mild/moderate recovered patients (**Figures 5B, C**). These results suggested that the expression of *HLA-DRA*, *HLA-DRB1*, *HLA-DPA1*, *HLA-DRB5*, *HLA-DQA1*, *HLA-DQB1*, and other HLA class II genes in monocytes and DCs in the recovery stage was related to the severity of COVID-19 disease, and their downregulation may contribute to the memory T cell differentiation–related transcripts inactivation and the low percentage of CD4m and CD8m T cells in severe/critical recovered patients compared to healthy controls.

## Discussion

The immune status of COVID-19 patients during the recovery stage is the key to whether they can obtain immune protection against SARS-CoV-2 reinfection. Basic immune memory and high serum antibody levels can be obtained about 28 days after foreign antigens invade the human body (36). Therefore, collecting peripheral blood of recovered patients 27–47 days after the onset of SARS-CoV-2 infection is one of the best choices to understand the immune characteristics of COVID-19 patients during the recovery stage, and find molecular markers related to disease severity or protection rate against reinfection. In this study, we generated single-cell sequencing data from the blood of SARS-CoV2 recovered patients and performed an integrated analysis with published data. We constructed a single-cell transcriptome landscape map of peripheral immune cells of recovered COVID-19 patients.

The results of the proportions and cytokine scores of cell subtypes in recovered patients in our study are consistent with those reported by Ren et al. (9). However, due to differences in cell population annotations and the number of samples included, the results of some cell subtypes cannot be directly compared. The samples we analyzed only included samples with a sampling time of 27–47 days (days after symptom onset). To determine if this impacted on results, we extracted sample data from the Ren dataset with 21–90 days sampling time (days after symptom onset) and assessed how the sampling time, severity, sample type, gender, and age influence cell subtype proportions (**Figure S2A**). We found gender has no significant effect on all cell subtypes, and age has significant effects on CD4m T, CD4m T (GZMK), CD8m (IL7R), prolif T, γδ T, and Vγ9Vδ2 T (**Figure S2A** and **Figure S3C**), all consistent with our subpopulation results. This shows that these findings were verifiable and stable in recovered COVID-19 patients for at least 2–3 months after symptom onset. Compared with Zhang et al.’s study on isolated T cells and B cells (8), our study obtained fewer TCR and BCR clones in each sample. Therefore, we have obtained very few characteristics of the T- and B-cell immune repertoire of recovered COVID-19 patients.

Our findings on HLA class II are different from those of Wilk et al. The Wilk et al. study focused on the progression stage of COVID-19 patients, while our study discusses the recovery stage. In the study of Wilk et al. (6), the HLA class II score was downregulated in patients with COVID-19 in the progression stage and the most decreased in severe/critical patients. In samples with sampling date 27–47 days after symptom onset, severe/critical recovered patients maintained lower HLA class II scores and higher ISG scores, compared to healthy control, while mild/moderate recovered patients were comparable to healthy controls. Furthermore, through cell–cell interaction analysis, we found several HLA class II genes, which are downregulated in severe/critical recovered patients but upregulated in mild/moderate recovered patients. These results suggested that the immune response of mild/moderate patients could gradually return to normal levels during the recovery stage, while severe/critical patients could remain in an immune disorder state. This might cause lower protection rate of severe/critical recovered patients against SARS-CoV-2 reinfection.

We also found that there was a higher proportion of CD14 monocytes in severe/critical recovered patients. These CD14 monocytes exhibited phenotypes such as upregulation of cytokine expression, downregulation of HLA class II genes, and antigen processing and presentation signaling pathway. Although the proportion of dendritic cells did not change, they also showed upregulation of cytokines and ISGs expression, and downregulation of HLA class II genes in severe/critical recovered patients. We observed a decreased proportion of CD4m T and CD8m T cells and showed a phenotype of downregulation of IL-17 and TNF signaling pathways in severe/critical recovered patients. This could suggest that the low antigen processing of dendritic cells and monocytes might negatively affect the memory T cell differentiation necessary to provide protection against SARS-CoV2 reinfection. The downregulation of HLA class II genes may be one of the reasons why convalescent patients with high serum anti-spike titers produced a higher proportion of non-neutralizing antibodies (15, 16, 18).

The proportions of most peripheral immune cell types in mild/moderate recovered patients were equivalent to that of healthy controls, but CD14 monocytes exhibited upregulated expression of inflammatory genes such as *TNF*. The expression of *HLA-DRA*, *HLA-DRB1*, and other HLA class II genes were also upregulated in CD14 monocytes and DCs. Similarly, T-cell differentiation regulation and memory T cell–related genes *FOS*, *JUN*, *CD69*, *CXCR4*, and *CD83* were upregulated. Therefore, we believe that the recovery of CD14 monocytes includes the return of cytokine expression and cell proportions to healthy levels. The upregulation of *HLA-DRA*, *HLA-DRB1*, and other HLA class II genes in CD14 monocytes and DCs may promote the change of CD4m T cell and CD8m T cell transcriptomes, helping the formation of T cell immune memory, thereby providing effective cellular immunity against SARS-CoV-2 reinfection. Further experiments are required to validate this hypothesis. Recent studies from Public Health England, London, UK, Andrews et al. found that the effectiveness of the Vaxzevria and Comirnaty vaccines against symptomatic diseases has greatly waned in people over 65 years of age (Unpublished, https://twitter.com/jburnmurdoch/status/1438100712441974786?s=19). This outcome seems to be similar to that found in SARS-CoV-2-infected people. This suggests that the differential immune characteristics we found in mild/moderate recovered and severe/critical recovered patients may be the keys to the development of an effective SARS-CoV-2 vaccine for the elderly.

SARS-CoV-2-specific memory T cell responses are long-lasting in recovered COVID-19 patients (37–39) even though SARS-CoV-2-specific antibody response may decrease (15, 37, 40–42). Previous studies have shown that T cell responses to SARS-CoV-1 and Middle East Respiratory Syndrome Coronavirus (MERS-CoV) are long-lasting, up to >17 years (43–45). Recent studies have also demonstrated that SARS-CoV-2-specific memory T cell response lasts for more than 10 months in recovered COVID-19 patients, and these cells are stem-like memory T cells with multifunctionality and proliferation ability (38). In addition, Katherine et al. found memory T cells contribute to protection against SARS-CoV-2 rechallenge in a rhesus monkey model (29, 30). These results support that the generation and persistence of memory T cells in recovered COVID-19 patients are essential for humans to prevent SARS-CoV-2 reinfection. Currently, there is no report on myeloid cell immunity in recovered patients 1 year or more after infection, and their role in preventing SARS-CoV-2 reinfection remains unknown. In addition, it is still unclear whether the T cell responses in severe COVID-19 patients who have recovered a year or longer after infection have returned to normal.

In summary, our analysis of a large-scale scRNA-seq dataset covering diverse disease severity has revealed multiple immune characteristics during the recovery stage of COVID-19 that have not been adequately studied previously. Such results provided a critical resource and important insights in dissecting the human body’s immune protection mechanism against SARS-CoV-2 reinfection and may help to develop effective SARS-CoV-2 vaccines for the elderly.

## Code Availability

Experimental protocols and the data analysis pipeline used in our work follow the 10X Genomics, Scanpy, Immunarch, and CellChat official documentation. The analysis steps, functions, and parameters used are described in detail in the **Methods** section. Custom scripts for analyzing data are available upon reasonable request.

## Data Availability Statement

The raw sequence data reported in this paper have been deposited in the Genome Sequence Archive of the Beijing Institute of Genomics (BIG) Data Center, BIG, Chinese Academy of Sciences, under accession code HRA000864 and are publicly accessible at http://bigd.big.ac.cn/gsa-human.

## Ethics Statement

The studies involving human participants were reviewed and approved by the Research Ethics Committee of the School of Public Health (Shenzhen) (2020-007), Sun Yat-sen University, China. Written informed consent to participate in this study was provided by the participants’ legal guardian/next of kin. Written informed consent was obtained from the individual(s), and minor(s)’ legal guardian/next of kin, for the publication of any potentially identifiable images or data included in this article.

## Author Contributions

YS designed the study. XL performed experiments, analyzed the data, and wrote the manuscript. ZM and MG analyzed the data and wrote the manuscript. QL, LY, ML, and ZW contributed to the data collection, data analysis, and data interpretation. HL, TJ, WW, and SF contributed to the samples collection and experiments. YS, ZM, and SF supervised the study and revised the manuscript. YB, NG, IP, and AB participated in revising and improving the English language of the manuscript. All authors contributed to the article and approved the submitted version.

## Funding

This work was supported by the National Natural Science Foundation of China (82041043) for YS, Shenzhen science and technology innovation program (kqtd20180411143323605 and JSGG20200225152008136) for YS, National Natural Science Foundation of China (8187631) for SF, Single Cell Gene Expression Atlas grant from the Wellcome Trust (no. 108437/Z/15/Z) and the Open Targets grant (OTAR2067) for ZM, EMBL predoctoral fellowship for MG.

## Conflict of Interest

The authors declare that the research was conducted in the absence of any commercial or financial relationships that could be construed as a potential conflict of interest.

## Publisher’s Note

All claims expressed in this article are solely those of the authors and do not necessarily represent those of their affiliated organizations, or those of the publisher, the editors and the reviewers. Any product that may be evaluated in this article, or claim that may be made by its manufacturer, is not guaranteed or endorsed by the publisher.
