# Supplementary material for: Single-Cell Analysis Reveals the Immune Characteristics of Myeloid Cells and Memory T Cells in Recovered COVID-19 Patients With Different Severities
Source: Front Immunol. 2022 Jan 3;12:781432. doi: 10.3389/fimmu.2021.781432 (PMC8762286; doi:10.3389/fimmu.2021.781432)

# Supplementary materials

## Figure legends for supplemental figures

**Figure S1. Basic characteristics of the integrated dataset and selected markers of cell subtypes.**

(A-B) Heatmaps of marker genes (bottom row) for multiple cell subpopulations. The left column and upper row present the cell subtypes identified based on combinations of marker genes.

(C) Boxplots depicting percentages of all cell types in PBMC cells, colored by group-specific color. T test with healthy, *p < 0.05, **p < 0.01, ***p < 0.001, ****p < 0.0001.

**Figure S2. The cytokine score, ISG score and multivariate correlation analysis of subtypes.**

(A) Heatmap for q values of ANOVA. Sample type, fresh or frozen; sample time, days after symptom onset. The range of PBMC samples included in the calculation is expanded to sample time 21-90 days (days after symptom onset) (Table S1).

(B) UMAPs of PBMC cells colored by cytokine score (top panel) and ISG score (bottom panel).

(C) Box plots show the cytokine score (top panel) and ISG score ​(bottom panel) of subtypes from healthy controls (n = 19), mild/moderate recovered (n=16), severe/critical recovered (n=6) patients. Significance was evaluated with T tests (and nonparametric tests), for each subtype versus healthy controls, *p < 0.05, **p < 0.01, ***p < 0.001, ****p < 0.0001.

(D) Dot plot depicting enriched signaling pathways in different serious groups in CD14 Mono (NFKBIA). Mono, Monocyte; HC, healthy controls; MR, mild/moderate recovered; SR, severe/critical recovered. The number in parentheses represents the number of genes with significant differences.

**Figure S3. Characteristics of the T and NK cells immune response in recovered COVID-19 patients.**

(A) Boxplots depicting percentages of T cell subtypes in PBMC cells, colored by group-specific color.

(B-C) The correlation analysis charts show the correlation between patient age and cell subtype proportions (Spearman’s correlation).

(D) Boxplots of the gene expression of CD8m T(GZMH), CD8m T(GZMK) and CD8m T(IL7R) cluster from healthy controls (n = 19), mild/moderate recovered (n=16), severe/critical recovered (n=6) patients. T tests (and nonparametric tests).

T tests (and nonparametric tests), *p < 0.05, **p < 0.01, ***p < 0.001, ****p < 0.0001.

**Figure S4. Effects of gender on BCR/TCR diversity, and the features of TCR and BCR repertoires.**

(A) Box plots show characterization and comparison of TCR clonal expansion of CD8m(GZMH) and CD8m(GZMK) among severe/critical recovered (SR) patients, mild/moderate recovered (MR) patients, and healthy controls (HC), by quantifying the percentage of expanded clones.

(B) Box plots show effects of gender on BCR/TCR diversity.

(C) Box plots show each V gene segment usage rate in each severity group.

(D) HCDR3 amino acid sequence length usage distribution bar graph.

T tests (and nonparametric tests), *p < 0.05, **p < 0.01, ***p < 0.001, ****p < 0.0001.

Figure S1.TIF


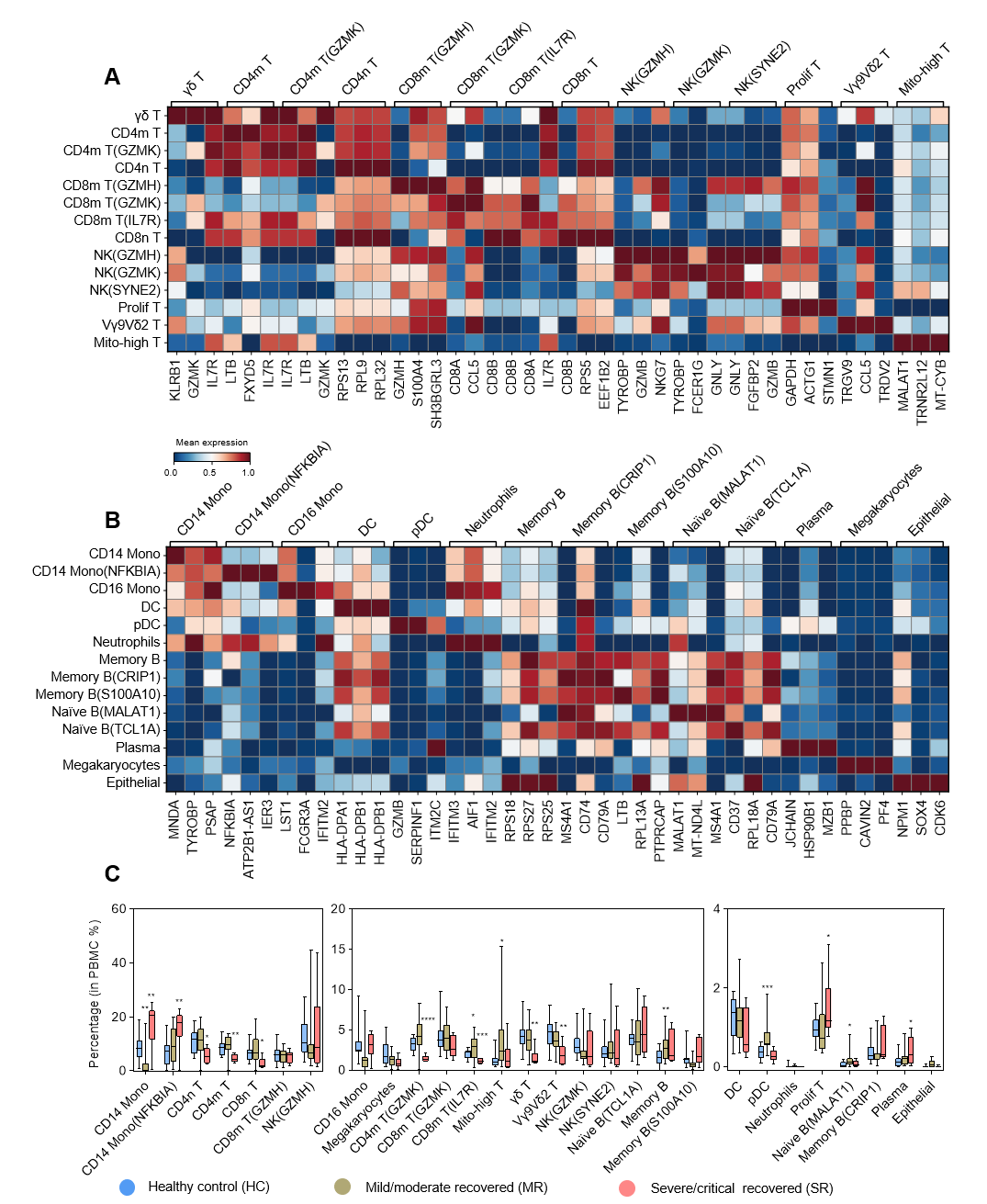


Figure S2.TIF


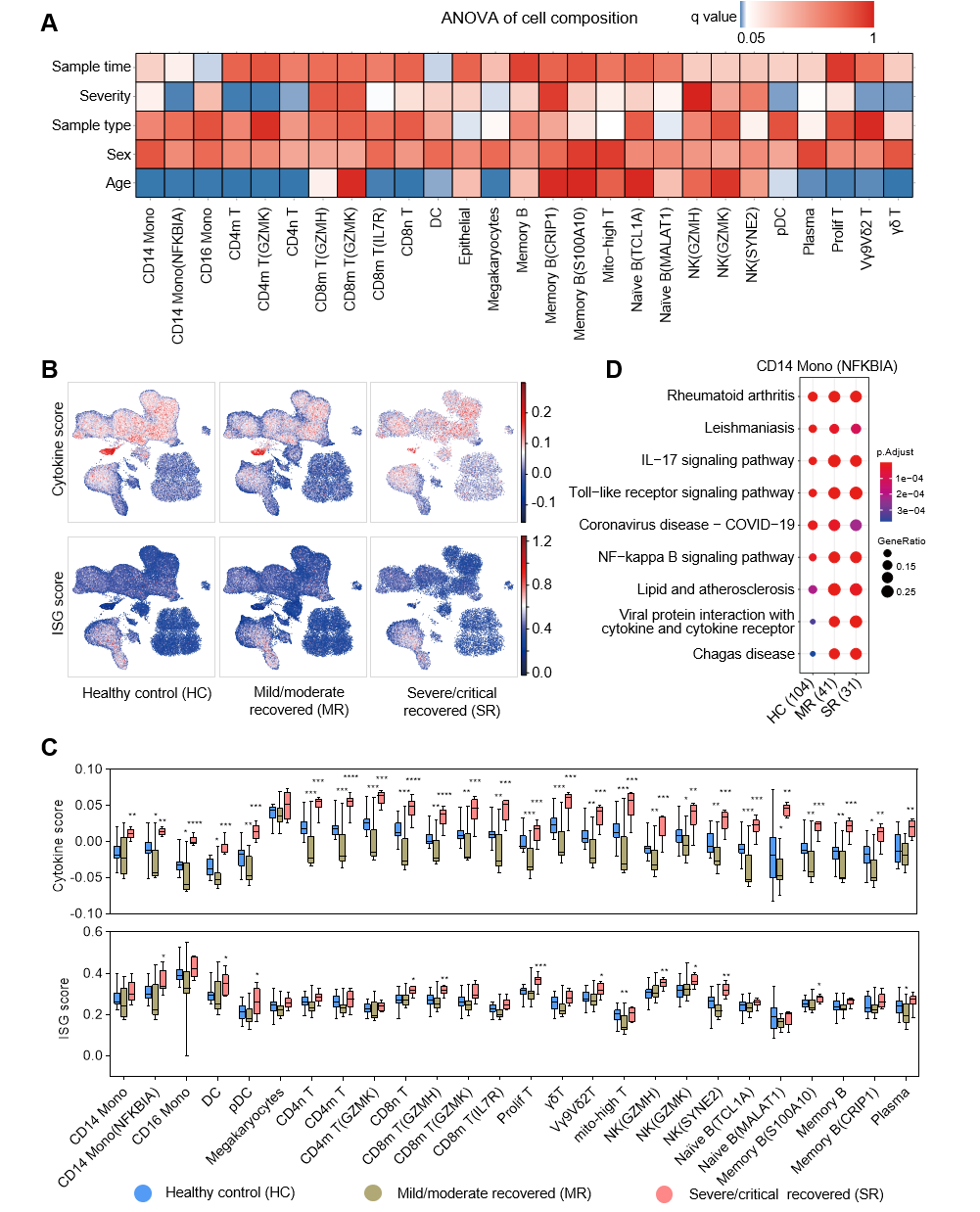


Figure S3.TIF


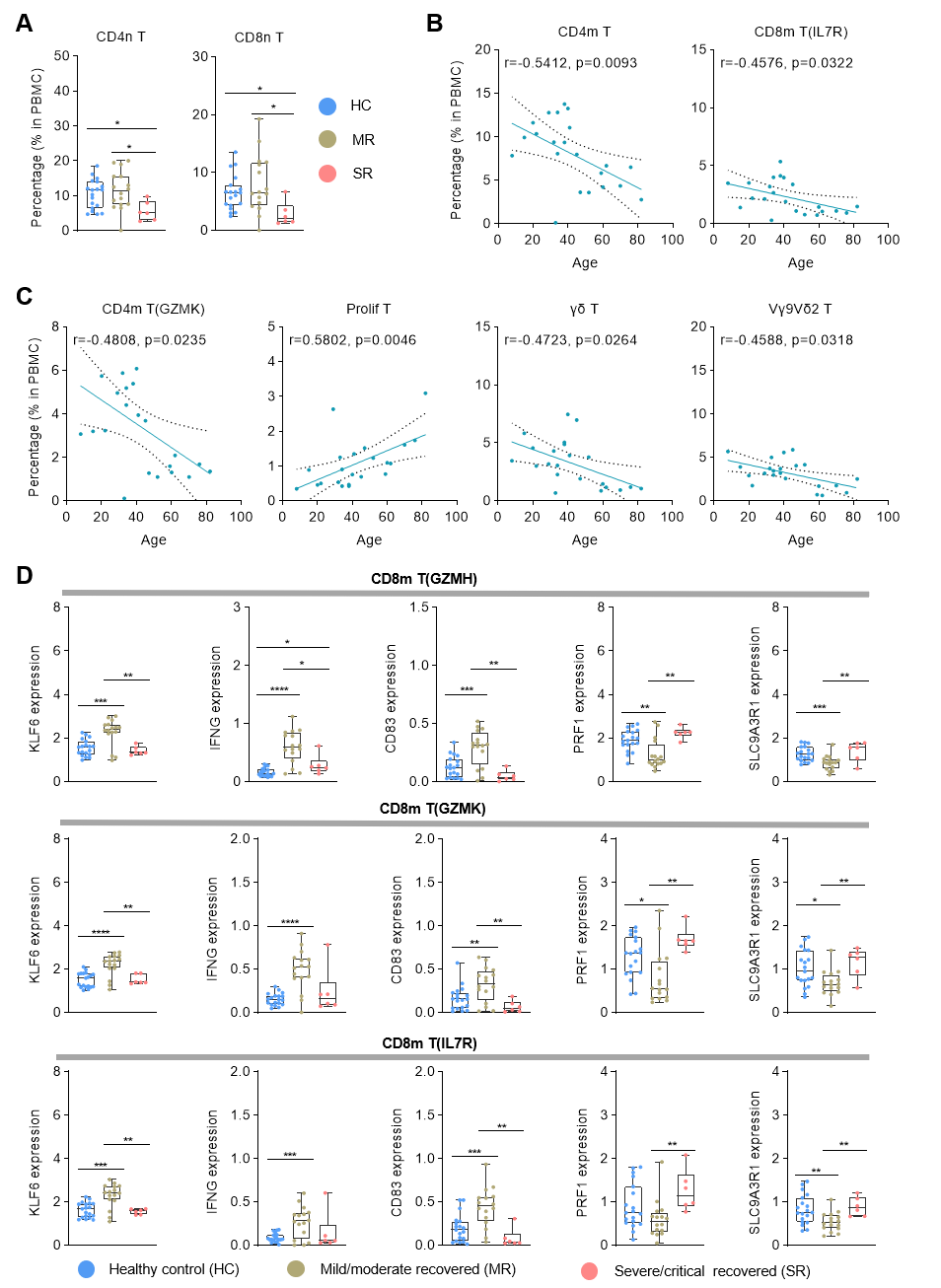


Figure S4.TIF


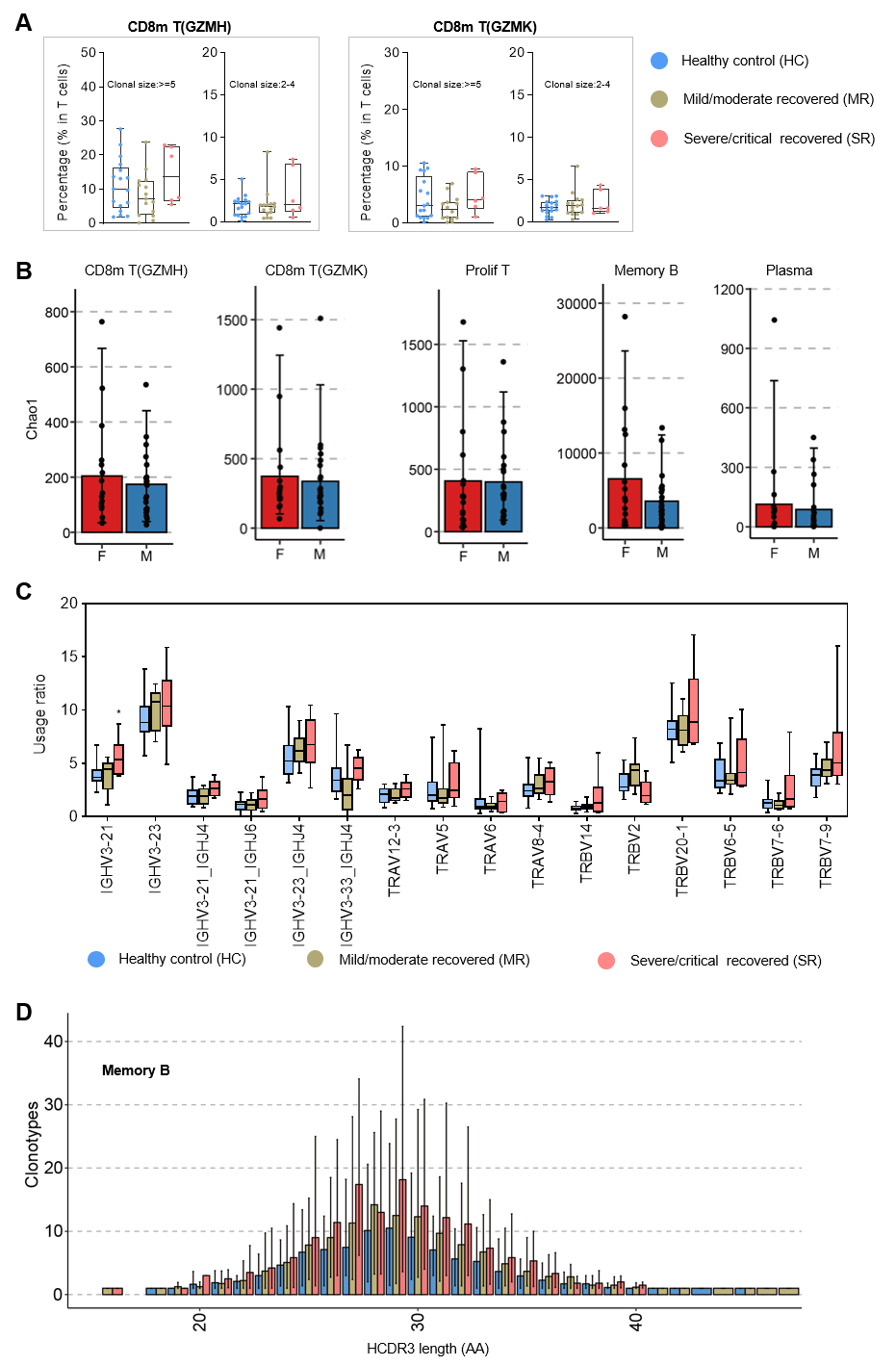

Supplement: Supplementary file 1 [file DataSheet_1.docx]
